# A Spontaneous Arterial Venous Fistula in a Patient on Hemodialysis: Case Report

**DOI:** 10.1002/ccr3.9640

**Published:** 2024-11-29

**Authors:** Getasew Kassaw Alemu, Addisu Melkie Ejigu, Beniam Yohannes Kassa

**Affiliations:** ^1^ Nephrology Unit, Department of Internal Medicine, College of Medicine Addis Ababa University Addis Ababa Ethiopia

**Keywords:** arteriovenous fistula (AVF), chronic kidney disease (CKD), spontaneous arteriovenous fistula

## Abstract

Arteriovenous fistula is an abnormal connection between arteries and veins occurring usually following surgery, trauma, catheterization, or local procedures. Spontaneous arteriovenous fistulas are a rare clinical entity with very few cases reported in the literature with unclear mechanisms. We present a case report of a 54‐year‐old Ethiopian female with known chronic kidney disease on hemodialysis for 2 years thrice a week who presented with progressive pulsatile right thigh mass of 7 months duration in the absence of trauma, catheter insertion previously, or surgery. The arteriovenous fistula was confirmed by computed tomography angiography and it was intervened with open surgery with no complication.

**Abbreviations**
AVF: arteriovenous fistula
CKD: chronic kidney disease
CTA: computed tomography angiography
HD: hemodialysis

**Summary**

- Spontaneous arteriovenous fistulas (AVFs) are a rare clinical condition with very few cases reported in the literature with unclear mechanisms.
- Spontaneous AVF can sometimes occur as a sequela of chronic kidney disease or hemodialysis but other common causes need to be excluded.

## 1 Introduction

Arteriovenous fistulas (AVFs) are a form of arteriovenous malformations clinically characterized by anomalous communications between arterial and venous systems that bypass the normal anatomic capillary beds [1]. Broadly it can be divided into two forms based on the cause: acquired or congenital. Surgery, penetrating trauma, and percutaneous catheterization are the most common causes of acquired AVFs. AVFs can be formed in the absence of a clear cause and are called spontaneous AVFs which are rare with very few case reports in the literature [2, 3, 4]. The anatomy of the fistula depends on the location in the body and where the causative factor was applied. Greater than 50% of traumatic AVFs happen in the lower extremity, and about one‐third occur in the femoral vessels, while 15% take place in the popliteal vessels but AVF can occur at any site [5, 6, 7, 8].

AVFs present clinically with progressively growing pulsatile mass on each respective site associated with or without complications including infection, thrombosis, aneurysm, hypertension, and heart failure demanding timely intervention [9].

## 2 Case Report

### 2.1 History and Examination

A 54‐year‐old female with end‐stage kidney disease on maintenance hemodialysis (HD) three times per week for the past 2 years after she was diagnosed to have pulmonary‐renal syndrome for which she has been getting prednisolone 5 mg orally daily since 2 years back. Two years back the patient was presented with hematuria and hemoptysis with elevated creatinine. She was put on high‐dose steroid (i.e., methylprednisolone 500 mg IV for 3 days followed by prednisolone 1 mg/kg which was gradually tapered). The pulmonary symptoms including cough with hemoptysis have improved with the prednisolone. She had an AVF on the left arm used for HD currently and the AVF on the right arm is not functional. She also had toxic nodular goiter and hypertension for the past 10 years. Currently, she is on amlodipine 10 mg orally per day, propylthiouracil (PTU) 100 mg twice per day, erythropoietin alpha 4000 IU SC weekly, and prednisolone 5 mg orally daily.

Her current presentation is progressively increasing pulsatile swelling over the right medial side of the mid‐thigh which progressed over 7 months with numbness on the same side; it concerns her as its size started to increase over the past 3 months but it has not bothered her for the first 4 months. She never had HD catheter inserted on the right femoral vein. There was no trauma, surgery, or venipuncture on the leg. There is no history of similar problems on another site. There is no history of smoking, alcohol drinking, or other substance use. There was no other chronic illness other than the mentioned ones. She had been treated for severe hospital‐acquired pneumonia (HAP) for 1 week after she had been admitted for intervention for which parenteral antibiotics were given for 7 days.

On physical examination: she is well‐looking and well‐nourished woman with blood pressure, heart rate, respiratory rate, and oxygen saturation of 130/80, 80%, 24%, and 96% respectively. Heart sounds were well heard and there was no murmur; there was a matured AVF with palpable thrill on the left cubital area and some on the right arm. There is an 8–9 cm length pulsatile mass on the medial aspect of the thigh with palpable thrill.

### 2.2 Investigation and Treatment

**FIGURE 1:**
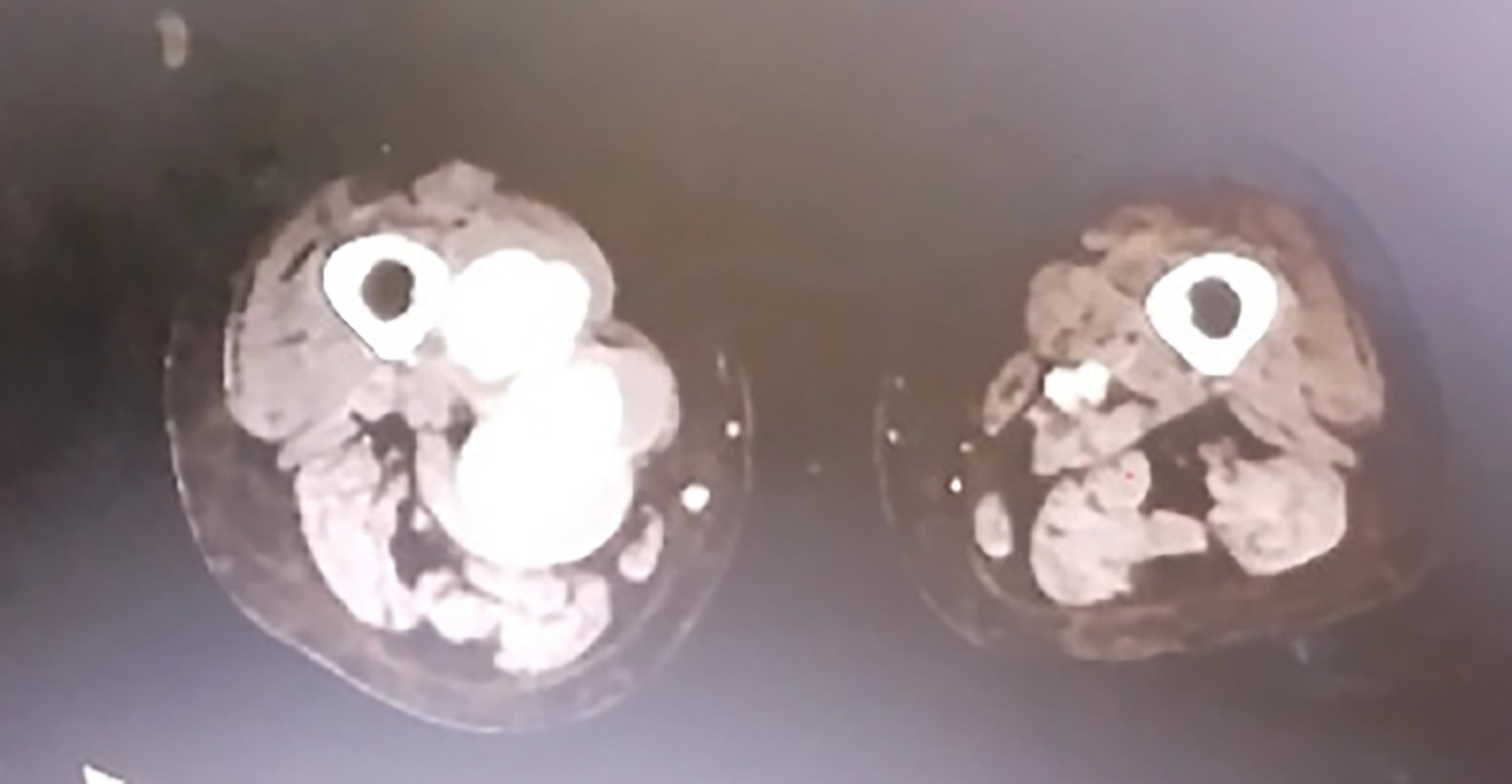
Right thigh CTA showing the aneurysm and AVF.

Complete blood count (CBC); Hgb ranges from 8.4 to 10 g/dL with MCV of 82: white blood cell and platelet count were within normal range. Her liver function test (LFT), serum sodium, potassium, chlorine, ionized calcium, and phosphorus are normal. Her stable creatinine is ranging from 4.5 to 5.5 mg/dL with eGFR < 10 mL/min/1.73 m^2^. Ferritin is 466 ng/dL. ANA test was negative. HIV test negative. Echocardiography shows mild concentric left ventricular hypertrophy (LVH) with preserved biventricular systolic function. Duplex arterial study of lower extremity report shows right middle superficial femoral artery (SFA) and superficial femoral vein (SFV) fistula with aneurysmal dilation and partial peripherally located thrombus. Computed tomography angiography (CTA) shows right SFA and SFV fistula with mild aneurismal dilatation at the AVF site (Figure 1).

After a diagnosis of spontaneous AVF was made, she was admitted to the vascular surgery ward while HD continued where the open surgical repair was done, the above medications continued (prednisolone was changed to hydrocortisone for 3 days).

### 2.3 Outcome and Follow‐Up

After the repair was done, the patient was discharged with improvement to continue the chronic follow‐up and HD. There was no other complication she developed during hospitalization.

## 3 Discussion

**FIGURE 2:**
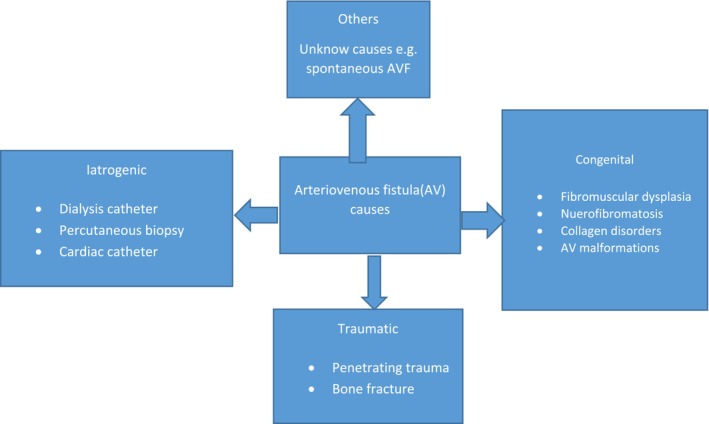
Causes of arteriovenous fistula; AVF—arteriovenous fistula.

Systemic AVF are rare but correctable vascular abnormalities usually occurring following catheterization procedures, surgery, trauma, or aneurysms; Spontaneous fistulas have occasionally been described [10]. There are cases reports of spontaneous AVF on different vessels in the absence of triggering cause, subclavian artery and vein [10], superficial femoral artery and vein [11], left common iliac artery and vein [12] and popliteal artery and vein [4]. The cause of spontaneous AVF formation may remain unknown and may need further study. Causes of AVF can be categorized irrespective of the vessel involved (Figure 2) for a simplified approach. Spontaneous AVF are rare in clinical activity with very limited reports in the literature but it is crucial to diagnose it promptly to prevent further complications including aneurysm, thrombus formation, and limb ischemia.

There has been a case report of spontaneous superficial femoral artery to femoral vein fistula in a 71‐year‐old female patient known to have multiple sclerosis in the background after her presentation with acute limb ischemia [11]. There is a report of AVF in the right lower limb following HIV arthritis [3]. Spontaneous right leg popliteal AVF had also been reported in a 79‐year‐old female where the cause remained unclear [4]. The patient presented in this article had chronic kidney disease (CKD) on the background of HD for 2 years. The clinical correlation between CKD and/or HD remains unknown but the CKD, HD, uremia, and vasculopathy genes may contribute to the formation of AVF through different mechanisms including vascular shear stress force, blood stasis, and cellular immunity dysfunction or their synergy [13, 14]. In conclusion, spontaneous AVF could occur as sequelae of CKD or HD but other common causes need to be excluded. Though there is a report of spontaneous AVF, generally AVF needs intervention [5, 6, 15].

## 4 Conclusion

Spontaneous AVF could occur as sequelae of chronic kidney disease or HD but other common causes need to be excluded.

## Author Contributions

**Getasew Kassaw Alemu:** conceptualization, data curation, formal analysis, investigation, writing – original draft. **Addisu Melkie Ejigu:** resources, supervision, validation, visualization, writing – review and editing. **Beniam Yohannes Kassa:** investigation, resources, writing – original draft, writing – review and editing.

## Ethics Statement

The institution does not require ethical approval for the publication of a case report and case series.

## Consent

Written informed consent was obtained from the patients. A copy of the written consent is available for review by the editor‐in‐chief of this journal.

## Conflicts of Interest

The authors declare no conflicts of interest.

## Data Availability

All data sets on which the conclusion of the cases for this study are based are available as a medical record document and are available from the corresponding author on reasonable request from the editor.
